# Bacterial Biodiversity of Extra Virgin Olive Oils and Their Potential Biotechnological Exploitation

**DOI:** 10.3390/microorganisms8010097

**Published:** 2020-01-10

**Authors:** Francesco Fancello, Chiara Multineddu, Mario Santona, Pierfrancesco Deiana, Giacomo Zara, Ilaria Mannazzu, Marilena Budroni, Sandro Dettori, Severino Zara

**Affiliations:** Dipartimento di Agraria, Viale Italia 39, University of Sassari, 07100 Sassari, Italy; fancello@uniss.it (F.F.); cmulti@uniss.it (C.M.); msantona@uniss.it (M.S.); pideiana@uniss.it (P.D.); gzara@uniss.it (G.Z.); imannazzu@uniss.it (I.M.); mbudroni@uniss.it (M.B.); sdettori@uniss.it (S.D.)

**Keywords:** *Bacillus* spp., *L. rhamnosus*, antimicrobial resistance, Extra Virgin Olive Oil (EVOO)

## Abstract

Bacterial diversity of 15 extra virgin olive oils, obtained from different Italian varieties, including Frantoio, Coratina, Bosana, and Semidana, was analyzed in this study. All bacterial isolates were genotyped using RAPD and REP-PCR method and grouped by means of cluster analyses. Sequencing of 16S rDNA of 51 isolates, representative of 36 clusters, led to the identification of *Bacillus* spp., *Brevibacillus* spp., *Micrococcus* spp., *Staphylococcus* spp., *Pantoea* spp., *Kocuria* spp., *Lysinbacillus* spp., and *Lactobacillus* spp., most of which reported for first time in olive oils. Phenotypic characterization of the 51 isolates, some of which ascribed to potentially probiotic species, indicate that two of them have beta-glucosidase activity while 37% present lipolytic activity. Preliminary evaluation of probiotic potential indicates that 31% of the isolates show biofilm formation ability, 29% acidic pH resistance, and 25% bile salt resistance. Finally, 29% of the isolates were sensitive to antibiotics while the remaining 71%, that include bacterial species well-recognized for their ability to disseminate resistance genes in the environment, showed a variable pattern of antibiotic resistance. The results obtained underline that microbial diversity of extra virgin olive oils represents an unexpected sink of microbial diversity and poses safety issues on the possible biotechnological exploitation of this microbial biodiversity.

## 1. Introduction

Over the last 15 years, microbiological research has established that freshly produced virgin olive oils have a rich microflora. Yeasts and bacteria are capable of conditioning the physicochemical and sensorial characteristics of the oil through their high enzymatic activities [1]. Certain yeasts are considered beneficial as they [2] can improve the sensorial characteristics of the oil during storage, whereas other yeasts are considered harmful as they can damage the quality of the oil through the hydrolysis of triacylglycerols and the production of unpleasant flavors [3,4,5,6,7,8,9]. The presence of these microbes, particularly the yeasts but also bacteria [9,10], possibly arises from their transfer into the oil from the olive carposphere during the extraction process. The microorganisms in olive oil are often below the limits of detection with standard culture methods [10] due to the strong selective pressure exerted by the oil’s antimicrobial compounds and the fact that the oil’s fatty acids constitute the sole source of carbon and energy for any microbial contaminates. These factors explain the scarcity of information about olive oil microflora. According to Koidis et al. [11], the presence of mini droplets of water in freshly produced olive oils support microbe survival in this hostile environment. Although the presence of microbes in olive oil may also result from manufacturing contaminations [12], a wealth of literature now indicates the presence of a highly specific microflora, especially in relation to yeasts [13]. By contrast, very limited data exist about the presence of bacteria. Recently, the groups of Santona et al. [9] and Pizzolante et al. [10] described the presence of different genera and species of bacteria in extra virgin olive oils that were at least one year old. While the effects of yeasts on the sensorial properties and quality of olive oils have been investigated in great depth [13], much less is known about the effects of bacteria. Olive oil microorganisms also possess great biotechnological potential thanks to their ability to tolerate and/or metabolize fats and greases in general; for these reasons, selected bacteria are frequently used for the bioremediation of oily wastewaters and contaminated soils or as a source of enzymes (e.g., lipases) or even biosurfactants [10]. Indeed, in this last cited study, the authors identified and characterized two bacteria strains of *Pantoea septica* able to produce carotenoids and bioemulsifiers. Another potential use of such bacteria is related to their probiotic characteristics, as recently demonstrated by Santona et al. [9]. Finally, it is important to recognize and stress that the presence of these microorganisms could also affect the safety of the olive oils due to the possibility of the bacteria possessing antibiotic resistance genes [14]. Considering that olive oil, due to its constituents, represents both a strong selective substrate for microbial growth microflora and a reservoir of microbial biodiversity, the aim of this work was to isolate, identify, and characterize bacteria from olive oils obtained from different national and regional varieties, in order to obtain bacteria with specific features for a potential biotechnological exploitation.

## 2. Materials and Methods

### 2.1. Isolation of Bacteria from Oil Samples

Olive samples were harvested (2016/2017 season) from an experimental olive grove belonging to the Department of Agriculture of the University of Sassari, situated in Oristano, Sardinia (Italy) (for more details see Deiana et al. [15]). Olive varieties used were: Frantoio, Coratina, Bosana, Semidana, Bianca di Villacidro, Confetto, Nera di Gonnos, Nera di Oliena, Palma, Paschixedda, Pizz’e carroga, Sivigliana da olio, Terza grande, Tonda di Cagliari, Tonda di Villacidro.

The production of the monovarietal olive oils was performed in an experimental mill, also located in Oristano, as described by Deiana et al. [16]. Bacterial isolation was performed under aseptic conditions according to Santona et al. [9] by using an enrichment method, with slight modifications. In brief, 10 ml oil was mixed with 90 ml YEPD (1% yeast extract, 2% peptone, and 2% glucose) in 250 ml Erlenmeyer flanged flasks and incubated with agitation for 24 h at 28 °C. Next, 100 μL aliquots were taken and 1/10 dilutions plated on six distinct culture media and maintained under different conditions as described below. Plate count agar (PCA, Merck, Italy) maintained at 28 °C for 48 h was utilized as the general medium for the viable mesophilic bacteria population, PCA maintained at 5 °C for 7 days was used for psychrophilic bacteria, PCA maintained at 45 °C for 48 h was used for thermophilic bacteria; and PCA was used for spore-forming bacteria treated at 80 °C for 15 min. MRS (De Man Rogosa Sharpe, Merck, Italy) agar was used to grow lactic acid bacteria at 30 °C, under both aerobiotic and anaerobiotic conditions (Thermo Scientific™ Oxoid AnaeroGen, Basingstoke, UK). Finally, YEPD and WL nutrient agar (Microbiol, CA, Italy) were utilized at 28 °C for 48 h for filamentous fungi, yeasts, and bacteria. All analyses were performed in triplicate. Randomly selected colonies, representative of the different colony morphologies (shape, color, dimension, halos, etc.), were manually picked up, re-streaked on BHI (brain heart infusion medium, Microbiol, CA, Italy), and stored at −80 °C until further analyses.

### 2.2. DNA Isolation, Cluster Analyses, and Molecular Characterization of Bacteria

Isolated bacterial colonies were inoculated into 15 mL sterile polypropylene tubes containing 7 ml BHI broth and kept overnight at 28 °C with agitation. The following day, samples were subjected to DNA isolation according to Santona et al. [9]. Next, in order to reduce genotypic redundancy among the strains, DNAs from every isolate were subjected to randomly amplified polymorphic DNA-polymerase chain reaction fingerprint analysis (RAPD-PCR), as described in Fancello et al. [17] by using the primer M13 (5′ GAGGGTGGCGGTTCT-3′) and REP-PCR as described by Mangia et al. [18] using the (GTG)5 oligonucleotide primer (5′-GTGGTGGTGGTGGTG-3′). For the molecular characterization, bacterial strains identified by RAPD-PCR were subjected to cluster analyses (InfoQuest version 4.5, Biorad), and strains representative of each cluster were chosen for further characterization. The DNAs of every representative strain were subjected to PCR of the 16S ribosomal DNA fragment (1500 bp) using the universal primers W001 (5′-AGAGTTTGATCMTGGCTC-3′) and W002 (5′-GNTACCTTGTTACGACTT-3′) [19]. Amplicons were then purified using the QIAquick PCR Purification Kit (QIAGEN GmbH, Hilden, Germany), following the manufacturer’s instructions, and sequenced. Sequencing was performed by Macrogen (Hong Kong, China). Sequencing was compared with those presented in the GenBank database using the BLAST program (http://www.ncbi.nih.gov/BLAST/) and with those in the Ribosomal Database Project (http://rdp.cme.msu.edu/edu/index.jsp). 

### 2.3. Enzymatic Characterization of Bacteria

All enzymatic tests were performed in triplicate and according to the methods reported in Santona et al. [9] with slight modifications. β-glucosidase activity was tested on agar plates with arbutin as substrate on YNB medium (6.7 g/L Yeast Nitrogen Base, Difco), plus 5 g/L arbutin (Sigma, St. Louis, MO, USA) and 20 g/L agar, at pH 5.0. Immediately after sterilization (121 °C for 15 min), 2 mL of a sterile 1% (*w*/*v*) ferric ammonium citrate solution was added to 100 ml of medium and then poured into Petri dishes (15 mL medium per plate). Each plate was inoculated with 8 different isolates, incubated at 25 °C, and examined after 2, 4, 6, and 8 days (d). A non-inoculated plate served as the control. Strains positive for β-glucosidase activity resulted in the development of a dark brown color in the agar due to hydrolysis of the substrate [20]. A code based on the color of the halo, indicative of the level of enzyme activity, was ascertained for each colony: White: N (no activity), light grey: W (weak activity), grey: M (moderate activity), and black: S (strong activity). 

β-glucanase activity was assessed by streaking the isolates onto brain heart infusion (BHI) plates containing 0.2% lichenan (Sigma). The plates were incubated for 5 d at 25 °C. The resulting colonies were then rinsed off the plates using distilled water and the plates stained with 0.03% Congo Red. A clear zone, around which a colony had been, identified β-glucanase activity [21]. A code based on the diameter of the halo and again indicative of the level of enzyme activity was allocated to each colony: N: No halo, W: Weak halo, M: Moderate halo, S: Strong halo.

Lipase activities were assessed on spirit blue agar (Sigma-Aldrich, St. Louis, MO, USA), which includes the pancreatic digest of casein (1%), yeast extract (0.5%), agar (1.7%), and spirit blue (0.015%). Lipase substrate mix contains Cottonseed oil (C7767, Sigma-Aldrich) and Tween 80 (Sigma, cat. 93,780). Lipase substrate was added to the medium following the manufacturer’s instructions. Plates were inoculated with the same amounts of cell suspensions and incubated at 25 °C for 48 h. Lipolysis was assessed by the presence of a halo around each colony (N: No halo, W: Weak halo, M: Moderate halo, S: Strong halo). 

For decarboxylase activity, agar plates were prepared as follows: Aliquots of 0.1 g/L glucose, 0.06 g/L of Bromocresol purple (Sigma- Aldrich), and 1 g/L of each amino acid (l-lysine, l-phenylalanine, L-tyrosine, L-histidine, and l-arginine) (used singularly and in combination) were added, and 15 g/L agar was suspended in 900 mL of distilled water. After sterilization, 100 mL filtered-sterilized yeast nitrogen base (Difco) solution (6.7% *w/v*) was added, adjusted to have a final pH 5.3. Isolates were streaked on the agar plates (of the medium prepared above) and then incubated at 25 °C for 4 d. The reaction was considered positive if a violet halo appeared around the colonies [22]. A code based on the color of the halo and indicative of the level of enzyme activity was ascertained for each colony: White: N, light grey: W, grey: M, and black: S. 

For catalase activity, isolates were inoculated into 96-well plates containing liquid BHI medium and incubated overnight at 30 °C. Enzyme activity was evaluated by adding, 2–3 minutes before the analysis, 3% (*v/v*) hydrogen peroxide to the inocula. The presence or absence of bubbles was indicative of the presence or absence of catalase activity, respectively, and the level of activity was once again graded accorded to the N, W, M, S code. 

### 2.4. Biofilm Formation

Biofilm formation was evaluated as described by Bou Zeidan et al. [23] and Santona et al. [9] with some modifications. In brief, 100 μL aliquots of cell suspensions containing 5 × 10^6^ cells/ml in M9 minimal salt medium (Gibco) were dispensed into 96-well polystyrene microtiter plates (Costar 3595, Corning, NY, USA). Cell suspensions were incubated statically at 25 °C for 24 h. The day after, 125 μL of the cell suspension and an equal volume of 0.1% (*w/v*) crystal violet were added to each well. After 30 min, the wells were washed three/four times with tap water and cell adherence quantified by solubilizing the retained crystal violet in 125 μL 30% CH3COOH for 15 min at room temperature. Next, 50 μL of these solutions was transferred to fresh 96-well polystyrene microtiter plates, and absorbance at 550 nm was measured spectrophotometrically. Classification of adherence capabilities was performed according to Extremina et al. [24]. The average optical density (OD) values were calculated for all tested strains and negative controls (nc), and the cut-off value (ODc) was established. ODc was defined as the average OD of the negative control (ODnc) plus three standard deviations of the negative control (SDnc), ODc = average ODnc + (3 × SDnc). Strains were divided into the following categories: OD ≤ ODc = non-biofilm producer, ODc < OD ≤ 2 × ODc = weak biofilm producer, 2 × ODc < OD ≤ 4 × ODc = moderate biofilm producer, 4 × ODc < OD = strong biofilm producer. 

### 2.5. Screening of Bacteria for Tolerance to Acidic pH and Bile

To assess tolerance to acidic pH, bacterial strains were inoculated into 24-well plates in liquid BHI and incubated at 25 °C overnight. The following day, cells were aspirated, and a concentration of 10^7^ cells/mL was then inoculated into fresh 24-well plates containing 1 mL liquid BHI adjusted to pH 2.5 with 12 N HCl and incubated at 37 °C for 2 h. Samples were removed after 2 h, and the viable strains were assessed by plating 10 μL of cell cultures on new BHI media and incubating the plates for 24 and 48 h at 25 °C.

To assess tolerance to bile salts, a 0.5% saturated bile solution was prepared by dissolving powdered bile salts (Fluka cod. #48305) in BHI broth and filter sterilized using a 0.2 mm filter. Bacterial strains were inoculated into 24-well plates in liquid BHI and incubated at 25 °C overnight. The following day, cells were aspirated, and 10^7^ cells/mL were inoculated into 24-well plates containing 1 mL of bile solution and incubated at 37 °C for 2 h. Wells with no bile solution were used as controls. Samples were taken after 2 h, and the viable strains were assessed by plating 10 μL of cell cultures on new BHI media and incubating the plates for 24 and 48 h at 25 °C. All screenings were performed in triplicate.

### 2.6. Antimicrobial Agent’s Susceptibility Test

The MICs of eight antimicrobial agents were determined by the use of broth microdilution methods according to the Clinical and Laboratory Standard Institute [25], the European Committee on Antimicrobial Susceptibility Testing (EUCAST; www.eucast.org), and ISO standards. In particular, plates for bacteria isolates were used containing serial 2-fold dilutions of the following antibiotics: ampicillin, ciprofloxacin, clindamycin, chloramphenicol, erythromycin, streptomycin, tetracycline, and vancomycin. For *Bacillus* spp., *Brevibacillus* spp., and *Lysinibacillus* spp., the MICs were determined by use of a modified Clinical and Laboratory Standards Institute (CLSI) method according to Agersø et al. [14]. The MIC of *L. rhamnosus* isolate was evaluated by the ISO 10932 standard, while the MICs of *Staphylococcus* spp., *Micrococcus* spp., and *Kocuria* spp. were evaluated according to CLSI guidelines for *Staphylococcus* spp. The MICs of isolates of *Pantoea* spp. were evaluated according to CLSI guidelines for Enterobacteriaceae. Epidemiological cut-off (ECOFF) values were retrieved from the European Food Safety Authority (EFSA) [26,27], from Agersø et al. [14]. For antibiotics not covered by EFSA, breakpoints from CLSI were used [25].

## 3. Results and Discussion

In our previous work [9], we made some first steps toward characterizing the bacterial microbiota of olive oil, whereas it constituted the primary goal of this present work, focusing on 15 different Italian olive varieties present in the experimental olive grove belonging to the University of Sassari. Through the isolation, molecular identification, and characterization of the microorganisms obtained from the different extra virgin olive oils analyzed, it was possible to elucidate, by using culture-dependent methods, the composition of the bacterial microbiota of the olive oils obtained. The total bacterial count was estimated using non-selective media (YEPD, WL, and PCA). Isolation of the oils’ microflora was performed using the enrichment method; thus, the estimates of concentration do not reflect the CFU/mL effectively present in the oils. Nonetheless, the CFU/ml concentration for bacteria present in olive oils subjected to enrichment was always an average of 5 × 10^3^ for all the oils tested. This is probably due to the strong selective pressure of many antimicrobial compounds present in the olive oils [28].

### 3.1. Molecular Characterization of the Isolates

In order to compare the genotypic redundancy among the strains isolated from olive oils, the RAPD and REP-PCR techniques were used. The fingerprinting methods used showed a good reproducibility with a very similar banding pattern amongst three independent DNA preparations of three biological replicates of the same isolate. In fact, the reproducibility of the electrophoresis pattern was 90%, which is the cut-off used for separating the isolates at strain level. The 72 isolates were grouped into 36 clusters with a cut-off value of 90%, 24 of which are formed by a single strain, proving the higher genotypic variability of the isolates from olives oils (Figure S1). No patterns were observed among the strains analysed. We did not find any linking between isolate origin and genotype of the strains. The 16S rDNA sequencing of the 52 isolated strains, obtained as representative of each cluster, revealed that the isolates mainly belonged to *Bacillus* spp., *Brevibacillus* spp., *Micrococcus* spp., *Staphylococcus* spp., *Pantoea* spp., *Lysinibacillus* spp., *Kocuria* spp., and *Lactobacillus* spp. In particular, among the *Bacillus* spp. we found *B. amyloliquefaciens* (11 strains), *B. subtilis* (5 strains), and *B. megaterium* (1 strain). Among *Brevibacillus* spp., we found *Br. agri* (9 strains), *Br. invocatus* (6 strains), and *Br. parabrevis* (3 strain). Among *Staphylococcus* spp. we found one strain of *S. pasteuri* and *S. epidermidis* and two strains of *S. hominis*. We also identified single strains of *Kocuria rhizophila* and of *Lactobacillus rhamnosus*. The other isolates were only identified at the genus level (4 strains of *Brevibacillus* spp., three strains of *Micrococcus* spp., three *Pantoea* spp., and one strain of *Lysinibacillus* spp.) (Table 1). As expected, most of the different genera and species found in the different olive oils analyzed are typical of natural environments, such as soils and plants, whereas some other genera/species identified are typical of the human host environment.

*B. subtilis* and *B. amyloliquefaciens* species are well-known for their plant growth-promoting rhizobacteria (PGPR) activity and have been widely used to promote plant growth and antagonize numerous plant pathogens [29]. For example, *B. amyloliquefaciens* is thought to promote plant growth through the biosynthesis of indole-3-acetic acid (IAA) molecules, enriching the available nitrogen, phosphate, and potassium in soil and improving soil health via a synergistic enhancement of several soil enzymes [30]. *B. subtilis*, on the other hand, is involved in the expression of specific genes and hormones, such as 1-aminocyclopropane-1-carboxylate deaminase (ACC) [31]. Of *Brevibacillus* spp., *Br. agri* strains have been found to tolerate and to be capable of degrading toluene [31], as well as able to reduce hexavalent chromium, preventing the generation of harmful byproducts [32]. A strain of *Br. invocatus* has been used for producing hydrogen from glucose [33], and *Br. parabrevis* has been studied for its ability to degrade low density polyethylene films (LDPE) [34].

We identified two strains of *Pantoea* at the genus level. *Pantoea* is a genus of gram-negative bacilli belonging to *Enterobacterales*, which may be beneficial or harmful to plants [35]. Recently, Pizzolante et al. [10] identified and characterized two strains of *P. septica* from olive oil that were able to produce carotenoids and bioemulsifiers, whereas Vuletin Selak et al. [36] sequenced the genome of a *Pantoea* spp. isolated from an olive knot. In olive knot disease, the nonpathogenic bacterial species *Pantoea agglomerans* and *Erwinia toletana*, both of which can live as epiphytes or endophytes, coexist with the pathogenic bacterium *Pseudomonas savastanoi* pv. *savastanoi*, the causal agent of knot disease [37,38,39].

Organisms in the genus *Kocuria* are environmental bacteria, as well as human skin and oropharynx mucosal commensals [40]. The actinobacteria *K. rhizophila* is gram-positive, halotolerant, and shows a high capacity to adapt to any specific ecological niche, being found in a large variety of environments, including marine sediment [41,42,43]. *K. rhizophila* has also been isolated from trout gut where it contributes to the physiological microflora [44], and it has been demonstrated to be an excellent example of an emerging pathogen in the context of this group, with proven pathogenic potential in salmonids [45]. *S. pasteuri*, on the other hand, has been isolated from an array of sources, including vegetables, goat milk, fermented pork sausages, and drinking water supplies. It has also been detected in the gastrointestinal tract of children with active celiac disease and in human vomit, urine, blood, and periprosthetic tissue [46,47]. *S. epidermidis* is a coagulase-negative *Staphylococci* and an important commensal organism of the human skin and mucous membranes. According to emerging evidence, its presence seems to convey benefits for human health through fighting harmful microorganisms. However, *S. epidermidis* can also be the cause of opportunistic infections, particularly biofilm-associated infections on indwelling medical devices [48]. Of the coagulase-negative staphylococci (CoNS), *S. hominis* is one of the three most frequently identified isolates recoverable from the blood of neonates and immunosuppressed patients and has been identified as a causal agent of bacteremia, septicemia, and endocarditis [49]. The presence of these *Staphilococci* species in the olive oil is almost certainly due to human contamination, probably due to the manual harvesting of the olives. *Micrococci* are gram-positive G+C-rich, non-motile, non-spore-forming actinomycetous bacteria. *Micrococcus* includes 10 members, with *Micrococcus luteus* being the type species. Members of this genus play important roles in the biodegradation of xenobiotics, bioremediation processes, and the production of biotechnologically important enzymes or bioactive compounds [50]. *Lysinibacillus* spp. belong to the family Bacillaceae. *Lysinibacillus* spp. have been found associated with plants but also isolated from air and natural soil habitats [51]. Finally, a strain of *L. rhamnosus* was found. *L. rhamnosus* belongs to the *Lactobacillus casei* group (LCG), which also includes the closely related *Lactobacillus casei* and *Lactobacillus paracasei*. LCG species are some of the most widely studied species due to their commercial, industrial, and applied health potentials [52]. Commercially, they are used to ferment dairy products. They have also been found to produce many bioactive metabolites able to confer host benefits when consumed [53]. As such, many LCG strains are considered probiotics. Among these, *L. rhamnosus* GG (LGG) is perhaps one of the most studied bacterial strains in relation to health applications [54]. 

It is important to underline that no pathogenic bacteria were isolated from the analyzed olive oils. This datum lies in agreement with that recently reported by Zullo et al. [12], who were unable to isolate any coliform bacteria from the different olive oils investigated and who concluded that any contaminating coliform bacteria must be destroyed in the oil mill during the malaxation of the paste that is usually richer in phenol compounds compared with the extracted olive oil. Considering the array of bacteria isolated in the present work, the malaxation effect described by Zullo et al. [12] is probably only relevant to coliform bacteria. In particular, as showed by Peng et al. [55], the presence of oleuropein in olive oil displays bidirectional activities in that it accelerates the growth of *Lactobacillus* strains but suppresses the growth of enteric bacterial pathogens. The presence of a strain of *L. rhamnosus* in one of the olive oils analyzed in this work supports the findings of Peng et al. [55], but we can also postulate that other bacterial species may have benefited from the presence of oleuropein and phenol compounds.

### 3.2. Enzymatic Tests and Biofilm Formation

Most of the bacteria isolated in this work had never previously been isolated from olive oils. All bacteria were tested to elucidate their enzymatic activities (β-glucosidase, β-glucanase, lipase, decarboxylase, and catalase) (Table 2). It is known that these activities are involved in the reduction of phenols and other olive oil molecules [6]. Most of the strains analyzed here showed very low β-glucosidase activity, except one strain of *B. amyloliquefaciens* and one strain of *Pantoea* that showed strong activity. Strong β-glucanase activity was exhibited by only one strain of *B. amyloliquefaciens*, two strains of *Brevibacillus* spp., one strain of *S. hominis*, and one strain belonging to *Pantoea* spp. Strong catalase activity was evident for two strains of *Br. agri*, one strain of *B. subtilis*, and one strain of *Bacillus* spp. Strong decarboxylase activity was shown by eight strains of *B. amyloliquefaciens*, four strains of *B. subtilis*, two strains of *Brevibacillus* spp., one strain of *Br. agri*, and one strain of *Micrococcus* spp. Finally, we found strong lipase activity in 10 strains of *B. amyloliquefaciens*, four strains of *B. subtilis*, two strains of *Br. agri*, one strain of *B. megaterium*, one strain of *Br. invocatus*, and one strain of *Brevibacillus* spp. All the other strains showed moderate, weak, or no enzymatic activities. As reported, the *Bacillus* spp. groups were the most active enzymatically. 

As regards the β-glucosidase activity, only two isolates showed this activity. In olive oil environment this is an important activity because the enzyme β-glucosidase degrades the oleuropein, the main phenolic compound present in olives, that gives an intense bitter taste [9]. The β-glucanase activity resulted positive in only five isolates. The β-glucanase hydrolyzes secoiridoid glycosides, with the releasing of antioxidant molecules [56]. Only four isolates showed peroxidase activity. This enzyme degrades the phenolic compounds and polyphenols and effects the sensorial qualities of olive oils [57]. As regards to the decarboxylase activity, 16 isolates showed this activity. The decarboxylation is also considered a negative activity because it leads to the production of biogenic amines able to trigger allergic and inflammatory reactions in humans [58].

Finally, it is important to emphasize the significance of the lipase activities displayed by some of the olive oil microorganisms isolated in this work. This activity has negative consequences in olive oil in terms of lipolytic activity [4] while being of great interest from a biotechnological standpoint. Indeed, microorganisms able to tolerate and/or metabolize oils can be used for the bioremediation of oily wastewaters and contaminated soils [10,59,60,61], and provide a source of biosurfactants [62,63]. Such bacteria may also be useful in other commercial sectors including the food, pharmaceutical, and cosmetics industries [63].

The ability of some microorganisms to form biofilms has generated much debate about the potential threat that these microorganisms, in this case bacteria, may pose to human health. In fact, while microbial adherence, in particular adherence to plastic or human tissues, is widely considered a threat for human health in terms of it providing a route to pathogenic contamination (for example, via indwelling medical devices such as urinary catheters), it is also seen as a highly positive trait, especially for probiotic microorganisms able to adhere to and persist in the gastro-intestinal tract [64]. Of the bacteria isolated in this work, the following showed strong biofilm formation ability: Eight strains of *B. amyloliquefaciens*, four strains of *B. agri*, one strain of *B. subtilis*, one strain of *Br. parabrevis*, and one strain of *L. rhamnosus* and *Pantoea* spp. Moderate ability was exhibited by two strains of *B. subtilis* and *Micrococcus* spp. and one strain of *B. invocatus, B. amyloliquefaciens, S. hominis, Pantoea* spp., and *Brevibacillus* spp. (Table 3). Considering these results, it is important to highlight the biofilm formation ability exhibited by the *Bacillus* spp. belonging to the PGPR, especially the *B. subtilis/B. amyloliquefaciens* group. Indeed, biofilms formed by this species in agricultural contexts confer important biocontrol properties [65]. In particular, it has been shown that many wild strains of B. subtilis are capable of forming biofilms on plant root surfaces and that biofilm formation increases cell colonization efficiency and enhances local concentrations of antibiotics that work as signal molecules and stimulate biofilm formation [66]. Finally, it is important to call attention to the ability of *L. rhamnosus* to form a biofilm. It is well documented that the ability to adhere to mammalian tissues constitutes a crucial feature that potential probiotic bacteria must exhibit if they are to adapt to the gastrointestinal tract [64]. The strain of *L. rhamnosus* isolated here (as described later on), however, showed no resistance to acid or bile salts.

### 3.3. Acid and Bile Salt Resistance of Bacterial Isolates

Acid and bile salt resistance tests provide good indexes of the ability of microorganisms to survive in the gastrointestinal tract. These microorganisms may be viewed positively if the specific isolates in question are being considered as potential probiotics or negatively if the microorganisms are identified as potential pathogens. Zullo and Ciafardini [1] recently showed that some olive-borne yeasts exhibit probiotic potential, but with the exception of our previous preliminary study [9], to the best of our knowledge this present study is the first to test bacteria isolated from olive oils for their probiotic potential. The following isolated bacteria showed strong acid resistance: Five strains of *B. amyloliquefaciens*, five strains of *B. subtilis*, and one strain of *Br. agri, Br. invocatus, S. hominis, Brevibacillus* spp., and *Pantoea* spp. Moreover, three strains of *B. subtilis*, two strains of *B. amyloliquefaciens, S. hominis, Pantoea* spp., and one strain of *Br. agri, K. rhizophila*, and *Micrococcus* spp. showed strong bile salt resistance (Table 4). As long as these isolates are not also characterized by negative enzymatic or potential pathogenic activities, their presence in the olive oils or their utilization in other food matrices could be seen as positive due to their probiotic potential. In this context, it is important to underline the recent interest being directed toward spore-forming bacilli used for many centuries for the production and preservation of food. Specifically, *Bacillus* spp. are receiving much attention in the field of functional food research due to their enhanced tolerance and ability to survive in the hostile environment of the gastrointestinal tract. Moreover, bacilli are highly stable during food processing and storage, making them highly suitable candidates for health-promoting formulations [67].

Thus, based on the results obtained, the probiotic candidature of the spore-forming *B. subtilis* and *B. amyloliquefaciens* isolated and characterized in this work may be considered. Last but not least, the isolation of a strain of *L. rhamnosus* was significant because, although this LCG showed no resistance to acid or bile salts, olive oil is a non-typical habitat for this species, making its presence there a noteworthy finding. 

### 3.4. Antimicrobial Resistance of Bacteria

Antimicrobial resistance is becoming a real problem due to the extensive use of antibiotics in both humans and animals. It has been estimated that antimicrobial resistance causes 25,000 deaths annually in the European Union and 23,000 in the US [14]. 

In this work, we tested all the isolates against a plethora of antibiotics chosen according to the recommendation by the European Food Safety Authority (EFSA) as main classes of antimicrobials employed in human and veterinary treatments. We obtained some remarkable results, as shown below. The species-related differences in the sensitivities of the strains to different concentrations of vancomycin, tetracycline, and chloramphenicol were observed among the three *Bacillus* spp. All the *Bacillus* spp. strains tested were susceptible to vancomycin (MIC range: 0.25–4 mg/L), tetracycline (0.5–16 mg/L), chloramphenicol (1–4 mg/L), erythromycin (0.25 mg/L), and ciprofloxacin (0.25 mg/L). Regarding clindamycin, all the *B. amyloliquefaciens* strains had MICs in the range of 0.5 to 1 mg/L, with a distribution of MIC values similar to that reported by Agersø et al. [14]. *B. subtilis* strains had MICs in the range of 2 to 4 mg/L, while the only strain of *B. megaterium* analyzed was resistant, in agreement with Agersø et al. [14], who found almost all *B. megaterium* strains to be resistant to clindamycin. The high resistance to clindamycin is probably, according to Adimpong et al. [68], an intrinsic characteristic of this species, as shown for *B. licheniformis*. Only one strain of *B. subtilis* and one strain of *B. amyloliquefaciens* were resistant to streptomycin, with MICs of 32 and 16 mg/L, respectively. In *B. amyloliquefaciens*, the resistance to streptomycin was attributed to the presence of a putative aminoglycoside 6-adenylyltransferase gene [14] with high similarity to the *aadk* gene that confers resistance to *B*. *subtilis* [69]. The MICs of other *Bacillus* isolates varied from 1 to 8 mg/L. Agersø and colleagues [14] proposed epidemiological cut-off values (ECOFF) for streptomycin of 2 and 8 mg/L for *B. megaterium* and *B. amyloliquefaciens* species, respectively, whereas the ECOFF value for streptomycin proposed by the European Food Safety Authority (EFSA) for *Bacillus* spp. in general is 8 mg/L. For ampicillin, our results confirmed those obtained by Agersø et al. [14]. 

All isolates of *Brevibacillus* spp. were susceptible to vancomycin, tetracycline, ampicillin, ciprofloxacin, and chloramphenicol. For clindamycin, differences in the sensitivities were noticed for the isolated strains of *Brevibacillus* spp., for which the MIC values ranged from 0.5–8 mg/L. Only one isolate of *B. agri* was susceptible, with an ECOFF valve of 4 mg/L, in line with the data published by the EFSA (2018) for *Bacillus* spp. The species showing the most sensitivity to clindamycin were the *Br. invocatus* isolates. Several *Brevibacillus* spp. isolates showed streptomycin resistance. Pawlowski et al. [70] recently revealed a correlation between the presence of a streptomycin 6-nucleotidyltransferase gene (ant(6)-Ic) in *Brevibacillus brevis* and sensitivity to streptomycin. One isolate of *Br. agri* was resistant to erythromycin. Pawlowski et al. [70] identified a macrolide kinase gene (*mphJ*) within the resistome of *Br. brevis* that heterologously expressed *mphJ* in *E. coli* and conferred resistance to erythromycin. Although this gene is only weakly expressed, it is feasible that it is involved in other metabolic processes in *B. brevis* VM4.

*Brevibacillus* spp. are occasionally used as biocontrol agents for promoting plant growth and protection from plant pathogens. In this regard, Pawlowski et al. [70] underlined that the utilization of antibiotics in agriculture may have resulted in the rapid dissemination of resistance genes from these genera into other plant pathogens and environmental bacteria. For these reasons, these genera should be carefully studied for their ability to mobilize resistance genes in areas/situations where the use of antibiotics is high. 

All *Staphylococcus* isolates were susceptible to vancomycin (MIC range: 0.5–4 mg/L) and chloramphenicol (2–8 mg/L); in contrast, they showed resistance to clindamycin (0.5–4 mg/L), and three out of the four strains isolated were resistant to tetracycline. Another study reported *Staphylococcus* spp. to be relatively susceptible to clindamycin [71]. The genetic determinants underlying the resistance of coagulase-negative staphylococci to clindamycin, erythromycin, and tetracycline were attributed to the presence of *erm(C)*, *mph(C)*, *erm(A)*, *tet(L)*, and *tet(K)* genes [72]. A multidrug resistance phenotype was observed in an isolate of *S. hominis* (resistance to six out of the eight antibiotics tested was observed). 

All *Micrococcus* isolates were susceptible to erythromycin and chloramphenicol, whilst being resistant to clindamycin. The *K. rizophila* isolate was susceptible to almost all the antimicrobial agents tested with the exception of ciprofloxacin, confirming the data of Savini et al. [41]. Although these seem to be reassuring, many authors have confirmed that *Kocuria* spp. is emerging as a human pathogen, in particular in compromised hosts with severe disease (see the review of Purty et al. [73]).

The one strain of *Lactobacillus* spp. isolated here, *L. rhamnosus*, has previously been shown by several authors to exhibit resistance to just a single antibiotic, vancomycin. In fact, *Lactobacillus* spp. demonstrate intrinsic resistance to this antibiotic, which is also the best characterized intrinsic resistance mechanism [74]. Finally, the two *Pantoea* spp. isolates identified were both resistant to erythromycin, ampicillin, ciprofloxacin, and streptomycin. One strain also demonstrated high resistance to clindamycin, vancomycin, and erythromycin. Finally, Agnew et al. [75] reported that *Pantoea* species isolates from Brent geese cloacal swabs can be considered opportunistic pathogens due to their antimicrobial resistance features, helped by their environmental dissemination due to the migratory movements of these birds. All data related to antibiotic resistance are summarized in Table 5.

## 4. Conclusions

In this preliminary work, we showed that in olive oil, which is considered an unfavorable substrate for microbial growth microflora, there are different bacterial species, and that both frequency and diversity of the bacterial species isolated depend on olive variety. Indeed, olive oil bacteria, together with yeasts, may influence chemical sensorial properties of olive oils during storage. However, they also represent a reservoir of microbial diversity that needs further consideration. On the one hand, some of the species found show an interesting biotechnological potential for industrial bioconversion of lipids, fats, and oils into high-value products and as plant growth-promoting rhizobacteria, chemical fertilizer substitutes, for their potential ability to detoxify industrial or agro-industrial byproducts. Moreover, *Bacillus* spp. and *L. rhamnosus* have a probiotic potential that may be explored in view of the possible probiotic utilization of some extra virgin olive oils. On the other hand, most of the isolates showed different patterns of antibiotic resistance, thus posing safety issues on the possible biotechnological exploitation of this microbial biodiversity.

## Figures and Tables

**Table 1:** Bacteria Isolates divided per species and olive oil varieties.

Strains	No. Isolates	Frantoio	Coratina	Bosana	Semidana	Bianca di Villacidro	Confetto	Nera di Gonnos	Nera di Oliena	Palma	Paschixedda	Pizz’e Carroga	Sivigliana	Terza Grande	Tonda di Cagliari	Tonda di Villacidro
*B. amyloliquefaciens*	11	-	-	-	-	9	1	-	-	-	-	-	-	-	-	1
*B. megaterium*	1	-	-	-	-	1	-	-	-	-	-	-	-	-	-	-
*B. subtilis*	5	-	-	-	-	1	-	-	-	-	-	4	-	-	-	-
*Br. agri*	9	1	-	-	2	-	-	2	1	1	-	-	2	-	-	-
*Br. parabrevis*	3	-	-	-	-	-	-	-	1	1	-	-	-	1	-	-
*Br. invocatus*	6	-	-	-	3	-	-	-	.	2	-	-	1	-	-	-
*L. rhamnosus*	1	1	-	-	-	-	-	-	-	-	-	-	-	-	-	-
*S. epidermidis*	1	-	-	-	-	-	-	-	-	-	1	-	-	-	--	-
*S. pasteuri*	1	-	-	-	-	-	-	-	-	-	-	-	-	1	-	-
*S. hominis*	2	-	-	1	-	-	-	-	-	-	-	-	-	-	1	-
*K. rhizophila*	1	-	1	-	-	-	-	-	-	-	-	-	-	-	-	-
*Brevibacillus* spp.	4	-	-	-	2	-	-	-	2	-	-	-	-	-	-	-
*Pantoea* spp.	3	-	-	-	3	-	-	-	-	-	-	-	-	-	-	-
*Lysinbacillus* spp.	1	-	-	-	1	-	-	-	-	-	-	-	-	-	-	-
*Micrococcus* spp.	3	-	-	1	1	-	-	-	-	-	-	-	-	-	1	-
Total	52															

Hyphen (-) indicates that none of the isolates of the specific species is present in a certain variety.

**Table 2:** Enzymatic activities of bacteria.

Strains	No. Isolates	Glucosidase				Glucanase				Lipase				Decarboxylase				Catalase			
		N	W	M	S	N	W	M	S	N	W	M	S	N	W	M	S	N	W	M	S
*B. amyloliquefaciens*	11	4	4	1	2	1	-	9	1	-	-	1	10	2	-	1	8	9	-	2	-
*B. megaterium*	1	-	1	-	-	1	-	-	-	-	-	-	1	-	1	-	-	-	1	-	-
*B. subtilis*	5	-	1	4	-	-	-	5	-	-	1	--	4	1	-	-	4	1	-	3	1
*Br. agri*	9	7	1	1	-	3	2	4	-	2	1	4	2	8	-	-	1	4	-	3	2
*Br. parabrevis*	3	3	-	-	-	3	-	-	-	-	1	1	1	-	3	-	-	2	1	-	-
*Br. invocatus*	6	4	2	-	-	5	-	1	-	-	1	4	1	2	6	-	-	6	-	-	-
*L. rhamnosus*	1	1		-	-	1	-	-	-	1	-	-	-	1	-	-	-	1	-	-	-
*S. epidermidis*	1	1	-	-	-	1	-	-	-	1	-	-	-	1	-	-	-	1	-	-	-
*S. pasteuri*	1	-	1	-	-	-	-	1	-	1	-	-	-	1	-	-	-	1	-	-	-
*S. hominis*	2	1	1	-	-	-	1	-	1	-	1	1	-	-	1	-	-	1	-	-	-
*K. rhizophila*	1	-	1	-	-	1	-	-	-	1	-	-	-	1	-	-	-	-	1	-	-
*Brevibacillus* spp.	4	3	1	-	-	2	-	-	2	2	-	1	1	2	-	2	2	3	-	-	1
*Pantoea* spp.	3	1	1	-	1	2	-	-	1	2	-	1	-	2	-	-	-	1	1	1	-
*Lysinbacillus* spp.	1	-	1	-	-	1	-	-	-	-	-	1	-	1	-	-	-	-	-	1	-
*Micrococcus* spp.	3	2	1	-	-	2	1	-	-	2	-	1	-	2	-	-	1	3	-	-	-

N: No activity; W: Weak activity, M: Moderate activity; S: Strong activity. Hyphen (-) indicates that none of the isolates of the specific species has a certain enzymatic activity level.

**Table 3:** Biofilm formation of bacteria.

Strain	No. Isolates	Adhesion to Plastic			
		No Biofilm	Weak	Moderate	Strong
*B. amyloliquefaciens*	11	-	2	1	8
*B. megaterium*	1	1	-	-	-
*B. subtilis*	5	-	3	1	1
*Br. agri*	9	1	3	1	4
*Br. parabrevis*	3	-	2	-	1
*Br. invocatus*	6	-	5	1	-
*L. rhamnosus*	1	-	-	-	1
*S. epidermidis*	1	-	1	-	-
*S. pasteuri*	1	-	1	-	-
*S. hominis*	2	-	1	1	-
*K. rhizophila*	1	-	1	-	-
*Staphylococcus* spp.	1	-	-	1	-
*Brevibacillus* spp.	4	-	3	1	-
*Pantoea* spp.	3	-	1	1	1
*Lysinbacillus* spp.	1	1	-	-	-
*Micrococcus* spp.	3	-	1	2	-

Hyphen (-) indicates that none of the isolates of the specific species has a certain adhesion level.

**Table 4:** Acidic and bile tests.

Strains	No. Isolates	pH 2.5				Bile Salt 1.5%			
		N	W	M	S	N	W	M	S
*B. amyloliquefaciens*	11	1	5	-	5	6	2	1	2
*B. megaterium*	1	1	-	-	-		1	-	-
*B. subtilis*	5	-	-	-	5	-	2	-	3
*Br. agri*	9	7	1	-	1	5	1	2	1
*Br. parabrevis*	3	2	-	1	-	1	2	-	-
*Br. invocatus*	6	5	-	-	1	6	-	-	-
*L. rhamnosus*	1	1	-	-	-	1	-	-	-
*S. epidermidis*	1	1	-	-	-	1	-	-	-
*S. pasteuri*	1	1	-	-	-	1	-	-	-
*S. hominis*	2	-	1	-	1	-	-	-	2
*K. rhizophila*	1	-	1	-	-	-	-	-	1
*Staphylococcus* spp.	1	1	-	-	-	1	-	-	-
*Brevibacillus* spp.	4	-	1	2	1	1	1	-	2
*Pantoea* spp.	3	1	-	-	1	-	-	-	2
*Lysinbacillus* spp.	1	1	-	-	-	1	-	-	-
*Micrococcus* spp.	3	3	-	-	-	2	-	-	1

N: Non-resistant; W: Weak; M: Moderate; S: Strong. Hyphen (-) indicates that none of the isolates of the specific species has a certain resistance level to pH or bile salt.

**Table 5:** Antibiotics suceptibility (MIC expressed in mg/L).

								MIC									
Strain Code	Species	CLI		VAN		ERI		TET		AMP		CIP		STR		CMP	
50	*B. amyloliquefaciens*	0.5		0.5		0.25		16		1	R	0.25		4		2	
52	*B. amyloliquefaciens*	0.5		0.5		0.25		16		0.5	R	0.25		2		2	
53	*B. amyloliquefaciens*	0.5		0.5		0.25		16		0.5	R	0.25		2		4	
54	*B. amyloliquefaciens*	0.5		1		0.25		16		1	R	0.25		8		2	
57	*B. amyloliquefaciens*	1		0.5		0.25		8		0.031		0.25		2		1	
58	*B. amyloliquefaciens*	0.5		0.5		0.25		16		>16	R	0.25		8		4	
75	*B. amyloliquefaciens*	1		1		0.25		16		4	R	0.25		4		2	
55B	*B. amyloliquefaciens*	0.5		0.5		0.25		16		2	R	0.25		2		4	
49	*B. amyloliquefaciens*	0.5		0.5		0.25		16		4	R	0.25		16	R	4	
113	*B. amyloliquefaciens*	1		0.5		0.25		16		0.5		0.25		4		2	
56	*B. amyloliquefaciens*	0.5		0.5		0.25		16		2		0.25		8		2	
61	*B. megaterium*	>16	R	0.25		0.25		0.5		8	R	0.25		1		4	
11	*B. subtilis*	2		1		0.25		4		0.031		0.25		32	R	2	
13	*B. subtilis*	4		4		0.25		4		0.031		0.25		2		4	
16	*B. subtilis*	4		4		0.25		4		0.062		0.25		4		4	
20	*B. subtilis*	4		4		0.25		4		0.062		0.25		4		2	
59	*B. subtilis*	0.5		0.5		0.25		16		8	R	0.25		4		4	
1	*Br. agri*	8	R	1		32	R	0.5		0.125		1		8		2	
3	*Br. agri*	2		0.5		1		1		0.125		1		16	R	8	
32	*Br. agri*	1		1		1		1		0.125		0.25		16	R	4	
34	*Br. agri*	2		0.5		1		2		0.031		0.25		8		1	
90	*Br. agri*	0.25		2		0.5		1		0.125		0.5		4		8	
79	*Br. agri*	2		0.5		0.125		0.5		0.031		0.05		16	R	8	
89	*Br. agri*	2		0.5		0.25		8		0.062		0.5		16	R	2	
112	*Br. agri*	2		0.5		1		2		0.031		0.25		16	R	2	
102	*Br. agri*	1		0,5		1		2		0.0625		0.25		8		1	
2	*Br. invocatus*	0.5		0.5		1		2		0.125		0.25		4		2	
29	*Br. invocatus*	2		0.5		0.5		2		0.062		0.25		16	R	1	
30	*Br. invocatus*	0.125		0.5		0.125		2		0.031		0.25		4		2	
35	*Br. invocatus*	0.125		0.5		0.125		2		0.031		0.25		4		2	
103	*Br. invocatus*	0.25		1		0.5		4		0.031		0.25		8		2	
108	*Br. invocatus*	0.25		0.5		0.125		2		0.031		0.25		4		2	
104	*Br. parabrevis*	4		1		0.5		2		8	R	0.25		64	R	4	
71	*Br. parabrevis*	0.25		4		>8	R	2		>16	R	0.5		4		>64	R
91	*Br. parabrevis*	0.25		1		0.5		2		0.031		0.25		8		1	
60TC2	*S. hominis*	1	R	0.5		1		0.125		0.031		0.25		16	R	2	
63B10	*S. hominis*	0.5	R	4		>8	R	>64	R	16	R	2	R	32	R	8	
70B	*S. pasteuri*	2	R	1		0.25		4	R	0.125		0.25		8		4	
73	*S. epidermidis*	4	R	0.5		0.25		2	R	0.031		0.25		2		4	
49C1	*K. rizophila*	0.125		2		0.125		0.25		0.031		2	R	1		2	
4	*L. rhamnosus*	0.25		>128	R	1		2		1		1		2		2	
221	*Lysinibacillus* spp.	8	R	1		8	R	2		0.25		2		32	R	4	
40B3	*Micrococcus* spp.	1	R	2		0.5		0.5		0.25		1		4		2	
60TC1	*Micrococcus* spp.	1	R	1		0.25		4	R	0.25		2	R	2		4	
225	*Micrococcus* spp.	4	R	32	R	1		1		8	R	0.25		8		4	
227	*Brevibacillus* spp.	2		1		0.125		0.5		0.031		0.25		4		4	
31	*Brevibacillus* spp.	1		0.5		1		2		0.031		0.25		16	R	2	
94	*Brevibacillus* spp.	4		4		0.25		4	R	0.062		0.25		2		4	
97	*Brevibacillus* spp.	4		0.5		0.25		4	R	0.062		0.25		4		4	
226A	*Pantoea* spp.	>16	R	>128	R	>8	R	>64	R	16	R	128	R	256	R	1	
226B	*Pantoea* spp.	1		2		>8	R	0.25		16	R	16	R	64	R	8	
223A	*Pantoea* spp.	2		0.5		0.25		1		0.062		1		8		16	

AMP: Ampicilin; VAN: Vancomycin; ERI: Erythromycin; TET: Tetracycline; CLI: Clindamycin; TR: Streptomycin; CIP: Ciprofloxacin; CMP: Chloramphenicol; R: Resistant. The epidemiological cut-off values, namely the MIC that separate a population into isolates with and without acquired or mutational resistance, were retrieved from the European Food Safety Authority (EFSA, 2012 and 2018) and from Agersø et al. (2018). For antibiotics not covered by EFSA, breakpoints from Clinical and Laboratory Standards Institute (CLSI) were used (CLSI, M100-S25, 2015). For *S. hominis, S. epidermidis* and *S. pasteuri*, the cut-offs of EUCAST (https://mic.eucast.org) were used. The values indicated represent the MIC of every single isolate when subjected to a range of different antibiotics at different concentrations, according to EFSA, CLSI, and the European Committee on Antimicrobial Susceptibility Testing (EUCAST). Unless otherwise stated with resistance (R), isolates are sensitive to the antibiotics.

## Supplementary Materials

The following are available online at https://www.mdpi.com/2076-2607/8/1/97/s1.
