# Supplementary material for: Bacterial Biodiversity of Extra Virgin Olive Oils and Their Potential Biotechnological Exploitation
Source: Microorganisms. 2020 Jan 10;8(1):97. doi: 10.3390/microorganisms8010097 (PMC7022595; doi:10.3390/microorganisms8010097)
Supplement: Supplementary file 1 [file microorganisms-08-00097-s001.pdf]

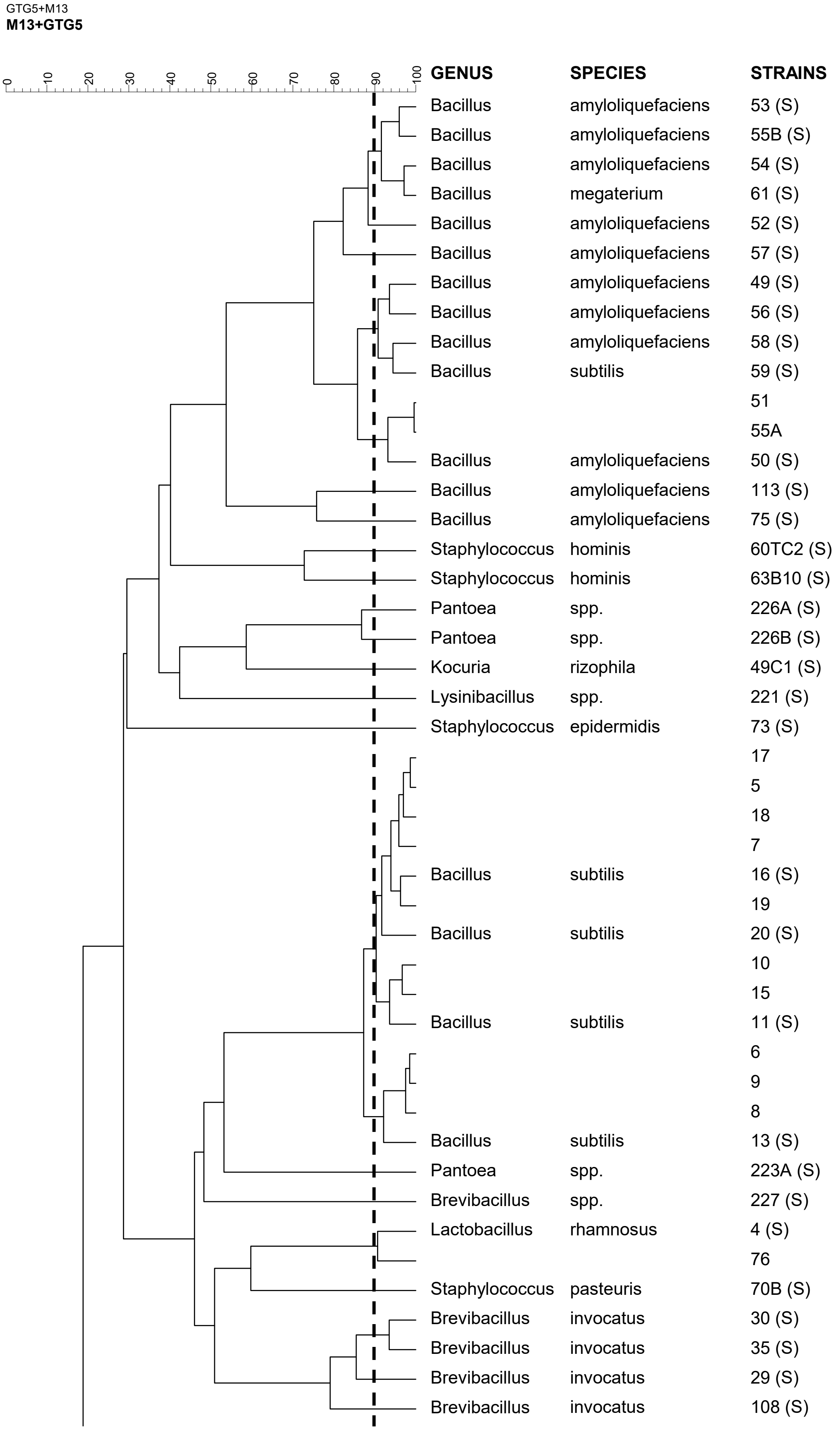
GTG5+M13
M13+GTG5
0 10 20 30 40 50 60 70 80 90 100
GENUS SPECIES STRAINS
Bacillus amyloliquefaciens 53 (S)
Bacillus amyloliquefaciens 55B (S)
Bacillus amyloliquefaciens 54 (S)
Bacillus megaterium 61 (S)
Bacillus amyloliquefaciens 52 (S)
Bacillus amyloliquefaciens 57 (S)
Bacillus amyloliquefaciens 49 (S)
Bacillus amyloliquefaciens 56 (S)
Bacillus amyloliquefaciens 58 (S)
Bacillus subtilis 59 (S)
51
55A
Bacillus amyloliquefaciens 50 (S)
Bacillus amyloliquefaciens 113 (S)
Bacillus amyloliquefaciens 75 (S)
Staphylococcus hominis 60TC2 (S)
Staphylococcus hominis 63B10 (S)
Pantoea spp. 226A (S)
Pantoea spp. 226B (S)
Kocuria rizophila 49C1 (S)
Lysinibacillus spp. 221 (S)
Staphylococcus epidermidis 73 (S)
17
5
18
7
Bacillus subtilis 16 (S)
19
Bacillus subtilis 20 (S)
10
15
Bacillus subtilis 11 (S)
6
9
8
Bacillus subtilis 13 (S)
Pantoea spp. 223A (S)
Brevibacillus spp. 227 (S)
Lactobacillus rhamnosus 4 (S)
76
Staphylococcus pasteuris 70B (S)
Brevibacillus invocatus 30 (S)
Brevibacillus invocatus 35 (S)
Brevibacillus invocatus 29 (S)
Brevibacillus invocatus 108 (S)

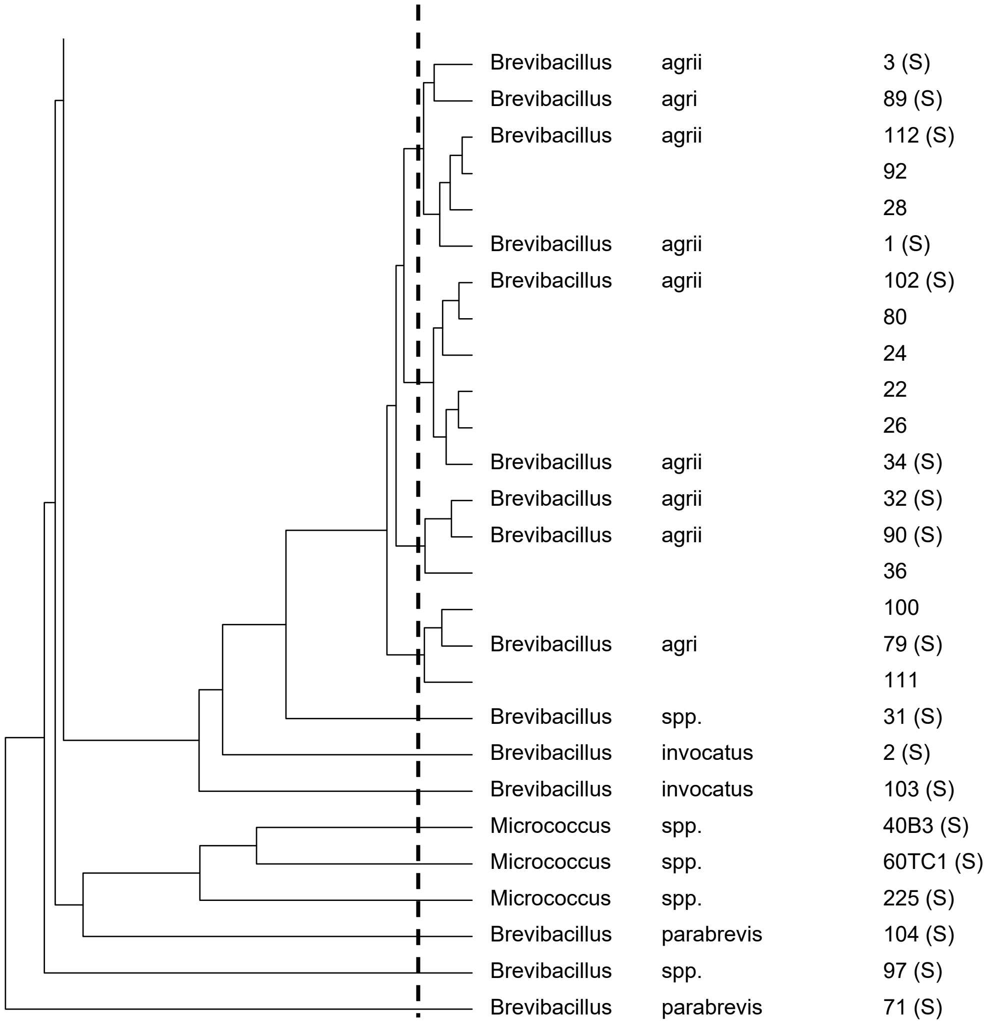

Figure S1. Genotyping by RAPD-PCR and REP-PCR of isolates from 15 olive oils. The unweighted-pair-group method with arithmetic averages (UPGMA) dendrogram is based on the Pearson correlation coefficient of the M13 and REP-PCR fingerprinting profiles. The dashed line indicates the cluster cut-off at 90 % similarity. The strains sequenced were also indicated by a S between brackets.
